# A Study on the Influence of Central Edge Absence in Helical Grinding for Micro-Hole Fabrication

**DOI:** 10.3390/ma17215260

**Published:** 2024-10-29

**Authors:** Bochuan Chen, Xiaojin Shi, Chong Zhang, Muhammad Amin, Songmei Yuan

**Affiliations:** 1 School of Mechanical Engineering and Automation, Beihang University, Beijing 100191, China; sxj_zy2307414@buaa.edu.cn (X.S.); yuansmbuaa@163.com (S.Y.); 2 Aerospace Research Institute of Materials & Processing Technology, Beijing 100076, China; zhangchongbuaa@163.com; 3 Department of Mechanical Engineering, Institute of Space Technology, Islamabad 44000, Pakistan; aminbuaa@yahoo.com

**Keywords:** micro-holes, helical grinding, central abrasive grain absence, disc-shaped residues, micro-PCD milling–grinding tool

## Abstract

The fabrication of micro-holes in hard-to-machine materials presents considerable challenges in precision machining. This study proposes a novel approach that employs high-strength micro-grinding tools with a central abrasive grain absence to create micro-holes through helical grinding. Due to the random distribution of abrasive grains, the absence of grains at the tool’s center becomes an inevitable technical challenge. This research examines the correlation between the diameter of the absence zone and the bottom morphology of the machined hole, highlighting the potential formation of disc-shaped or cylindrical residues. A model for predicting the height of the disc-shaped residues is developed, and the mechanisms governing their removal during grinding are further explored. The findings indicate that when a central grain absence exists, the first abrasive grain surrounding the absence zone, referred to as the inner-edge grain, is responsible for removing the disc-shaped residues. Based on these results, a novel 0.8 mm diameter micro-PCD milling–grinding tool with a central edge absence is designed, and experimental validation is performed using 65% SiCp/Al composite materials. The experimental results confirm that the central grain absence leads to the formation of disc-shaped residues at the bottom of the machined hole during helical grinding, and the morphology of the experimentally obtained residues aligns with the theoretical predictions and simulations. This study significantly advances micro-grinding wheel technology and provides a solid foundation for the precision machining of micro-holes in hard-to-machine materials.

## 1. Introduction

Ceramic materials exhibit a multitude of superior properties, such as high hardness, high weather resistance, and high temperature resistance, and they have rapidly increased their applications across various industrial domains [1]. In recent years, the development of non-conductive ceramic materials like zirconia [2,3], silicon dioxide [4], and silicon carbide fiber-reinforced silicon carbide [5,6] has progressed, alongside a growing demand for the machining of micro-holes [7,8] and the microstructures for such materials. Given the poor conductivity of composite ceramic materials, electrical discharge machining (EDM) is not feasible [9]. Meanwhile, the ultrafast laser machining of micro-holes is compromised by poor quality [10], with limited control over cylindricity [11], restricting the quality and aspect ratio of micro-holes [12]. Micro-grinding tools have demonstrated certain technological advantages for the machining of micro-holes in non-conductive ceramic materials.

Traditionally, the manufacture of micro-holes in ceramic materials has predominantly employed grinding tools with axial feed grinding [13,14], supplemented by peck drilling for chip removal [7]. However, due to the high hardness, strength, and brittleness of composite ceramics, peck drilling generates excessive axial forces, causing ceramic fracture and abrasive grain layer crushing [15]. As an alternative, helical feed grinding significantly reduces the undeformed chip thickness at the same cutting depth by extending the tool path through tool rotation [16], thus controlling the axial force [17,18]. This method allows for the machining of micro-holes with larger aspect ratios (≤5) and provides exceptional technical advantages, such as the direct drilling of holes on sloped or arced surfaces.

The diameters of the grinding wheels used in micro-hole helical grinding range from 0.3 to 0.8 mm, with the abrasive grains (30–80 μm) being relatively large and randomly distributed on the tool surface [19]. Constraints inherent to the manufacturing of such micro-grinding tools customarily result in a central absence of abrasive grains. This phenomenon is a substantial challenge as the sintering density of wheel grains has a theoretical upper limit [20,21]. It is therefore imperative to ascertain the effect of a central grain absence on the end face of the wheel in helical grinding.

Diamond grinding tools produced through different manufacturing techniques exhibit variations in the grain size, concentration and binder characteristics [22]. Research on the distribution of effective abrasive grains on grinding wheels primarily relies on experimental observation methods [23], including microscopic observation, roll printing, laser displacement sensing, and thermocouple temperature measurement [24]. However, due to technical limitations, the latter three methods are unsuitable for studying the effective abrasive grains on the wheel’s end face. Therefore, microscopic observation is a suitable method to ascertain the distribution of effective abrasive grains on the wheel’s end face.

Presently, the research on material removal processes through helical drilling predominantly involves kinematic analyses of the tools. The methodology/findings of research conducted by Brinksmeier et al. [25] and Denkena et al. [26] achieved remarkable acceptance for milling kinematics and helical grinding. The contemporary studies on the material removal process and cutting force modeling in helical milling/grinding [27,28] build upon these foundational works. Further studies have been carried out on material removal processes in helical milling using milling cutters with specially configured bottom edges [29,30]. The studies above all generally assume that the abrasive grain on the grinding wheel (or edge of the milling tool) is complete, with the cutting edges passing through the tool’s center [31]. Nonetheless, there remains a gap in the literature concerning the principles and material removal processes specific to helical grinding when there is an absence of central abrasive grains on the tool’s end face.

Building on prior research, this study investigates the impact of central grain absence in the helical grinding process. This paper first examines the prevalence of abrasive grain absence at the tool’s end face. A kinematic analysis is then conducted to determine that cylindrical and disc-shaped residues form at the bottom of the hole when the central grains are missing, and an analytical expression for the height of disc-shaped residues is derived. This study also analyzes the variations in the removal process of disc-shaped residues based on changes in the protrusion height of the bottom-edge abrasive grains and predicts the maximum height of such residues under different processing parameters. Finally, a multi-blade micro-PCD milling–grinding tool with a central edge absence is designed, and small-hole machining experiments are conducted on SiC_p_/Al (65% volume fraction) material. The results confirm that disc-shaped residues are present during machining when the central grains are absent, and a comparison of the experimentally measured maximum residue height with the theoretical and simulation results validates the accuracy of the proposed theoretical model.

## 2. Undeformed Chip Model with Central Abrasive Grain Absence

### 2.1. Central Grain Absence on Micro-Grinding Tool

**Figure 1:**
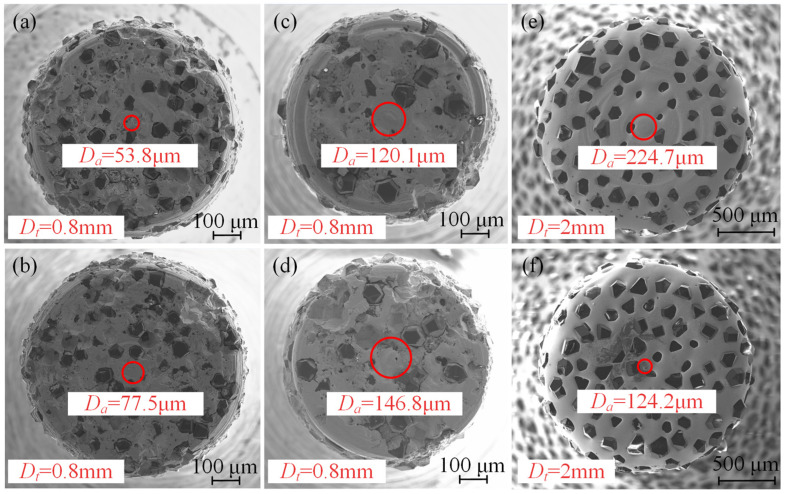
Central abrasive grain absence in the tool: (**a**,**b**) 0.8 mm diameter, 400# mesh sintered diamond abrasive tool; (**c**,**d**) 0.8 mm diameter, 140# grit sintered diamond abrasive tool; and (**e**,**f**) 2 mm diameter, 100# mesh electroplated diamond abrasive tool.

Micro-abrasive tools consist of a tool substrate, abrasive grains, and a bonding phase. The manufacturing processes include electroplating, sintering, and brazing, with diamond or cubic boron nitride (CBN) used as abrasive grains. Due to the decreasing circumference as the tool diameter reduces from the edge to the center, combined with the random distribution of abrasive grains across the tool’s end face, the probability of abrasive grains being deposited at the center decreases. This often results in a central grain absence zone. Regardless of the tool diameter or grain size, the formation of this absence zone is generally unavoidable. As shown in Figure 1, micro-abrasive tools produced through different manufacturing processes and with varying diameters consistently exhibit this absence zone at the center. For smaller diameter tools, the cutting forces they can withstand are limited. Therefore, it is essential to investigate the material removal process and mechanisms under conditions of central abrasive grain absence in helical grinding. Such research is crucial for reducing the grinding forces, extending the tool life, and enhancing the machining performance.

### 2.2. Basic Kinematic Parameters

**Figure 2:**
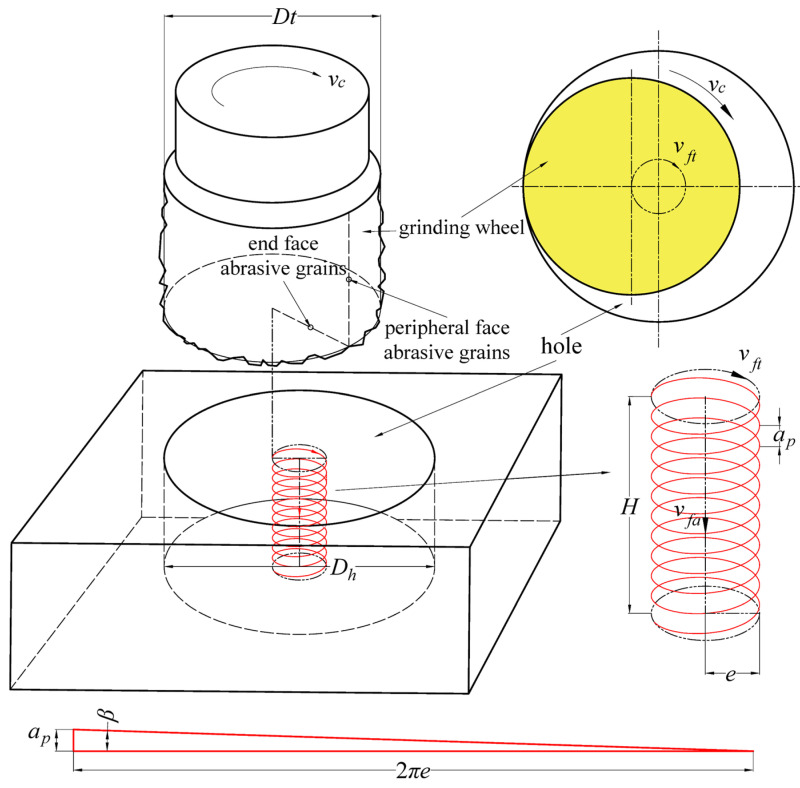
Geometric parameters in the helical grinding process.

The main parameters of helical grinding are illustrated in Figure 2. For analysis, it is assumed that the grinding tool rotates and orbits clockwise in a plane perpendicular to the tool’s axis. During machining, the tool rotates at high speed while orbiting at a certain eccentricity. The orbiting speed matches the machine’s horizontal feed speed (*v_ft_*, in mm/min), and its rotational speed aligns with the spindle speed (*n*, in r/min). The cutting speed (*v_c_*, in m/min) is combined with horizontal motion and axial motion (*v_fa_*, in mm/min), which keeps a constant speed, and *v_fa_* typically represents the single-layer cutting depth (*a_p_*, in mm) in the cutting program, indicating the axial advance per orbit. Additional parameters include the eccentricity (*e*), the diameter of the hole being machined (*D_h_*), and the diameter of the grinding tool (*D_t_*), with their geometric relationship being as shown in Equation (1).

(1) $$e=\frac{D_{h}-D_{t}}{2}$$

In helical grinding, the helix angle (*β*) is determined by the relationship between *a_p_* and *e*, as depicted by the displacement triangle shown in Figure 2, where the single-layer cutting depth *a_p_* is equivalent to the pitch. According to the principle that the horizontal helical period equals the axial feed period, the following expression can be found:

(2) $$\frac{2\pi e}{v_{ft}}=\frac{a_{p}}{v_{fa}}$$

### 2.3. Single Rotation Undeformed Chip Morphology with Central Grain Absence

**Figure 3:**
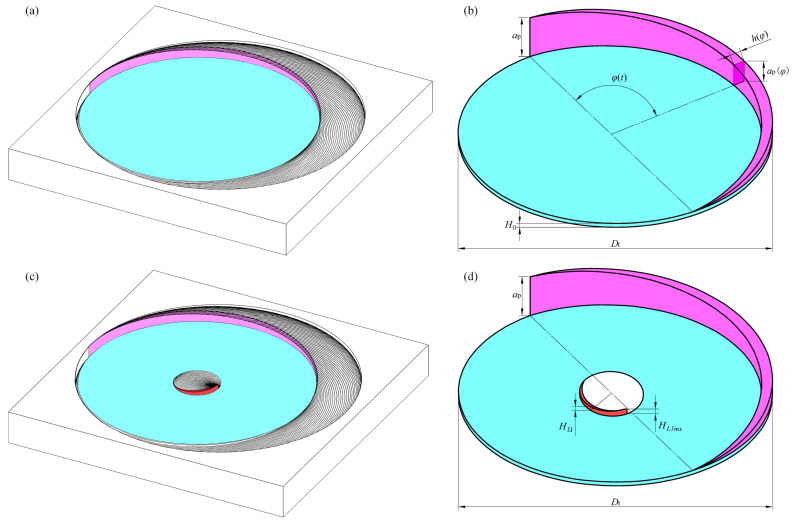
Morphology of the hole bottom and undeformed chips: (**a**) hole bottom morphology in the ideal condition; (**b**) undeformed chips in the ideal condition; (**c**) hole bottom morphology with a central abrasive grain absence; and (**d**) undeformed chips with a central abrasive grain absence.

Figure 3a shows the idealized bottom morphology in helical grinding, assuming a uniform distribution of effective grains on the tool’s end face without gaps [25]. Figure 3b presents the extracted undeformed chips, illustrating the ideal morphology. The single rotation undeformed chips from the side edge are shaded in magenta, with their thickness *h*(*φ*) and height *a_p_*(*φ*) being functions of the contact angle *φ*(*t*). The undeformed chips from the end face edge are represented as a blue-shaded disc, with thickness *H*_0_ and diameter *D_t_*. Figure 3c displays the bottom morphology in helical grinding when there is a central grain absence. Figure 3d shows the morphology of the undeformed chips in this condition, with the inner edge’s single rotation undeformed chips shaded in red.

As a central abrasive grain absence zone exists on the grinding tool, both the hole bottom morphology and the undeformed chip undergo significant changes. The hole bottom morphology can result in either disc-shaped residues or a combination of disc-shaped and cylindrical residues depending on the conditions described below.

**Figure 4:**
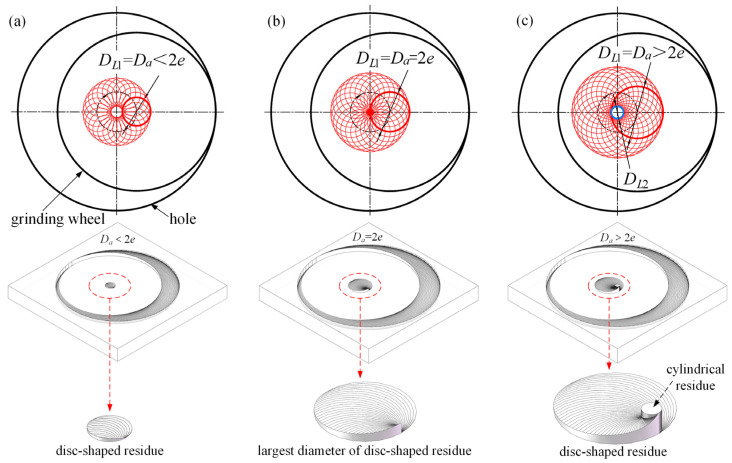
The relationship between the abrasive absence zone and the residues at the bottom of the hole: (**a**) disc-shaped residues with *D_a_* < 2*e*; (**b**) largest diameter of disc-shaped residues at *D_a_* = 2*e*; and (**c**) disc-shaped and cylindrical residues with *D_a_* > 2*e*.

For *D_a_* < 2*e* (Figure 4a), material in the grain absence zone is removed by the first effective grain (hereinafter referred to as the first grain) around the *D_a_*, and we can see it as the inner edge, resulting in a disc-shaped residue of diameter *D_L_*_1_:

(3) $$D_{L1}=D_{a}$$

For *D_a_* = 2*e* (Figure 4b), this condition results in the absence zone’s maximum allowable size; the disc-shaped residue’s boundary tangents the hole’s center, forming the largest diameter without creating cylindrical residues under similar conditions to *D_a_* < 2e.

For *D_a_* > 2*e* (Figure 4c), both disc-shaped and cylindrical residues appear at the hole’s bottom. The diameter of the cylindrical residues, *D_L_*_2_, can be determined through Equation (4). Such residues cannot be removed by any grain. If the workpiece has low strength or hardness, or if it is a hard–brittle material, the cylindrical residue is directly crushed by the tool’s end face, generating significant axial force and additional stress, forming debris. If the workpiece possesses high strength and hardness, the tool base is instead cut by the cylindrical residue. Hence, cylindrical residues are detrimental and must be avoided during machining.

(4) $$D_{L2}=D_{a}-2e$$

In the presence of disc-shaped residues, as depicted in Figure 3c,d, the grinding morphology and undeformed chip morphology vary. The red-shaded area represents the single rotation undeformed chips formed along the inner edge, generated by the first grain’s cutting action through the disc-shaped residue, with the magenta and blue-shaded areas indicating the peripheral and end face edge’s single rotation undeformed chips, respectively. The height of the inner edge of the single rotation undeformed chips corresponds to the disc-shaped residue’s height. To study the material removal process, the heights of both the cylindrical and disc-shaped residues are required to be found.

### 2.4. Height of Cylindrical Residues

The maximum height of cylindrical residues is determined by the characteristics of the grinding tool. When the grinding tool’s bottom features a central opening similar to a core drill, the height of the cylindrical residue equals the depth of cut made; if the cutting depth exceeds the opening’s depth, the cylindrical residue will press against the opening’s end, eventually causing tool failure.

**Figure 5:**
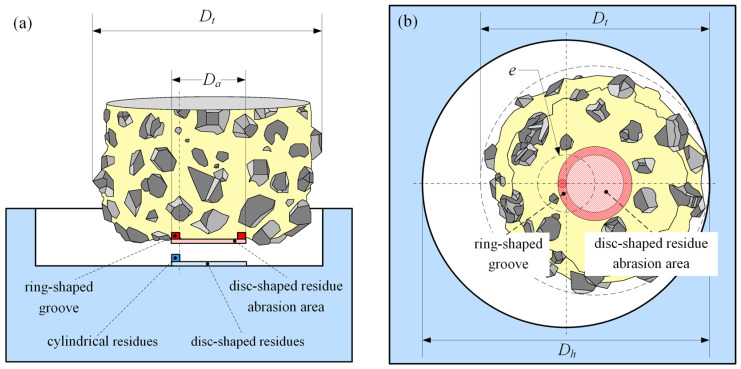
Mechanism of formation for cylindrical and disc-shaped residues: (**a**) side view, and (**b**) top view.

When the bottom of the grinding tool is fully closed, with only the abrasive grain absence present, as shown in Figure 5, the cylindrical residue will cut into the base of the tool’s end face in reverse, forming a ring-shaped groove whose width is defined by the cylindrical residue and whose maximum diameter is *D_a_*, depicted in the figure by a dark red ring. Simultaneously, the disc-shaped residue may also cause reverse erosion to the end face, but this depends on the relationship between the tool parameters and the cutting parameters, and this will be discussed in the following section. The maximum height of the cylindrical residue is determined by the depth of the ring-shaped groove. However, at any time, the height of the cylindrical residue is greater than or equal to the height of the disc-shaped residue.

### 2.5. Height of Disc-Shaped Residues

**Figure 6:**
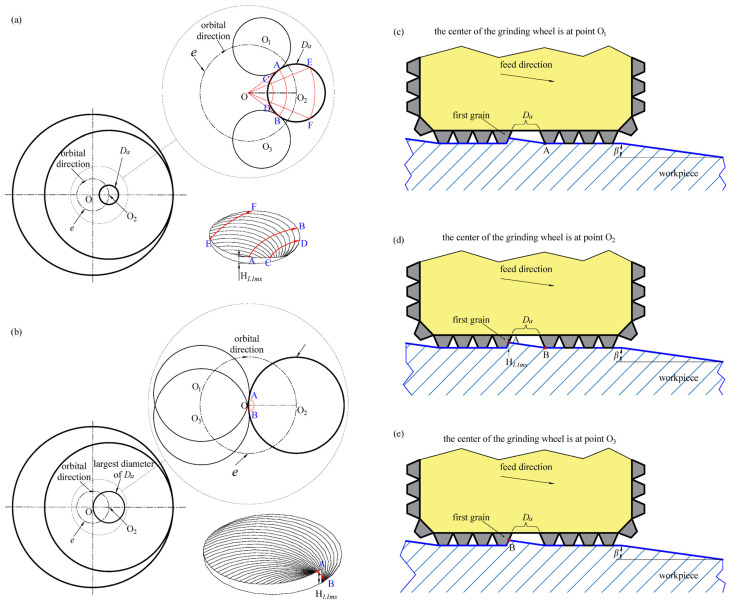
Formation process of disc-shaped residues: (**a**) morphology and formation process of disc-shaped residues; (**b**) morphology and formation process of the largest disc-shaped residues; (**c**) side view when the center of the tool is at O_1_; (**d**) side view when the center of the tool is at O_2_; and (**e**). side view when the center of the tool is at O_3_.

Figure 6 illustrates the material removal process with an abrasive grain absence at the center of the tool. During the tool’s orbital process, as shown in Figure 6a, its geometric center sequentially arrives at points O_1_, O_2_, and O_3_. At position O_1_, the first grain cuts point A with its end face, as shown in Figure 6c. Subsequently, point A remains within the absence area, unable to be processed by any grain, resulting in the continuous accumulation of residual height at point A until the center of the tool orbits to point O_2_, accumulating to its maximum height. At this point, the first grain cuts point A with its side edge, and after a half-turn, it similarly cuts point B for the first time, as depicted in Figure 6d. This interaction creates a significant height difference between points A and B, denoted as *H_L_*_1*mx*_. Afterward, point A starts being cut by other grains on the end face and its height gradually decreases. As the tool continues its orbit to position O_3_, point B achieves the same maximal residual height *H_L_*_1*mx*_, initiating a repeating cycle. Therefore, the position of the disc-shaped residue shifts dynamically with the orbital center of the tool, although its morphology remains constant.

As illustrated in Figure 6a, the maximum central angle corresponding to the orbital path is ∠AOB. Excluding points A and B, the central angles for the other points on the disc-shaped residue’s perimeter are smaller than ∠AOB, as observed in arcs CD and EF. Hence, contact point A corresponds to the highest position of the disc-shaped residue, denoted as *H_L_*_1*mx*_. Point B corresponds to the lowest position of the disc-shaped residue, with its height being zero.

When the absence zone’s diameter nears twice the eccentricity value, as shown in Figure 6b, the location of the maximum height difference shifts. When the absence zone’s diameter nearly equals twice the eccentricity, points A and B nearly converge at point O. The area around point A remains the highest point of the disc-shaped residue, and the height at point B stays at zero. At this time, a significant height variation occurs within a very short distance along arc AB, resulting in the formation of the largest diameter of the disc-shaped residue, termed the “largest disc-shaped residue”, as depicted in Figure 6b.

**Figure 7:**
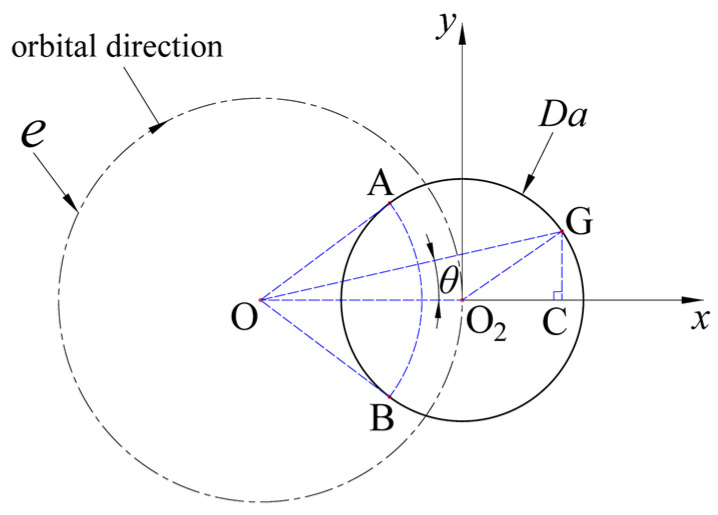
Derivation of the maximum height of the disc-shaped residues.

It is known that the orbital and axial movements of the grinding tool are consistent in the helical grinding process; thus, their velocity ratio remains constant [25,26]. When the tool moves from point A to point B, the ratio of the helical feed arc length to the circumference of the helical path equals the ratio of the axial feed depth to the single-layer cutting depth (*a_p_*). To ascertain the maximum height of the disc-shaped residues (*H_L_*_1*mx*_), calculating the central angle ∠AOB in Figure 7 is essential, as it determines the helical feed arc length and subsequently enables the derivation of *H_L_*_1*mx*_. As illustrated in Figure 7 and forming an *x*-*y* coordinate axis with O_2_ at the origin, given BO_2_ = *D_a_*/2, OO_2_ = *e*, and O_2_C = *x*, the value of angle ∠GOC, or *θ*, can be deduced through geometric relations:

(5) $$\theta =\arctan\frac{GC}{OC}=\arctan\frac{\sqrt{\frac{D_{a}^{2}}{4}-x^{2}}}{e+x}$$

The height of the disc-shaped residues at any point G, *H_L_*_1_, equates to:

(6) $$H_{L1}=\frac{2\theta }{2\pi }a_{p}=\frac{a_{p}}{\pi }\arctan\frac{\sqrt{\frac{D_{a}^{2}}{4}-x^{2}}}{e+x}$$

Considering the *x* values range from (−*D_a_*/2, *D_a_*/2), deriving the above equation yields:

(7) $$\frac{d\left(H_{L1}\right)}{dx}=\frac{a_{p}\left(-ex-\frac{D_{a}^{2}}{4}\right)}{\pi \left(e^{2}+2ex+\frac{D_{a}^{2}}{4}\right)\sqrt{\frac{D_{a}^{2}}{4}-x^{2}}}$$

Setting Equation (7) to zero, effectively making the numerator zero, identifies the *x* value corresponding to the maximum height of the disc-shaped residues.

(8) $$x=-\frac{D_{a}^{2}}{4e}$$

Incorporating Equation (8) into Equation (6) provides *H_L_*_1*mx*_’s formula:

(9) $$H_{L1mx}=\frac{a_{p}}{\pi }\arctan\frac{D_{a}}{\sqrt{4e^{2}-D_{a}^{2}}}$$

### 2.6. Grinding Removal Process for Disc-Shaped Residues

The cutting removal process for the disc-shaped residues can be divided into two types based on the protrusion height of the first grain.

Type 1: The protrusion height *h_i_* of the first grain is greater than the maximum height *H_L_*_1*mx*_ of the disc-shaped residue, with the disc-shaped residue being removed by the first grain.

**Figure 8:**
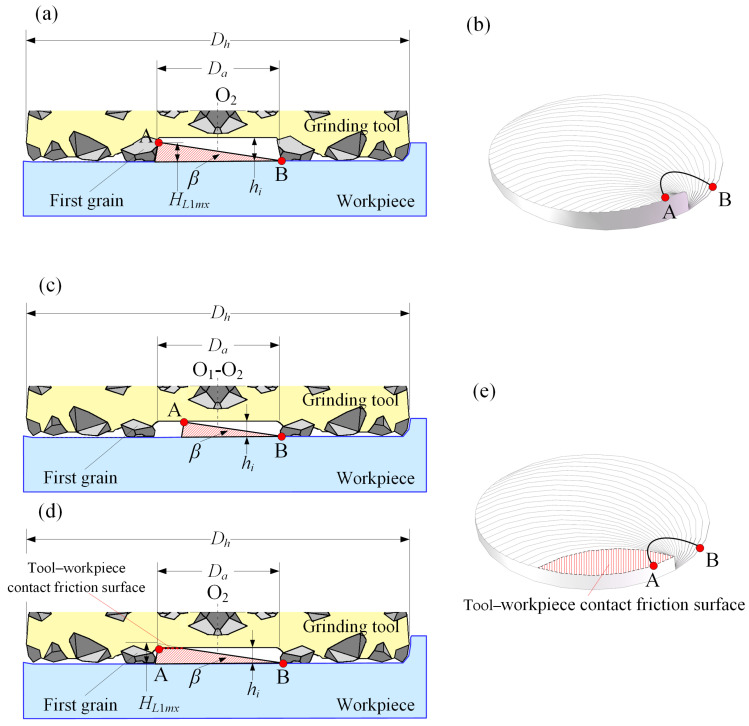
Grinding removal process of the disc-shaped residue: (**a**) removal process of the disc-shaped residue; (**b**) morphology of the disc-shaped residue; (**c**) the moment point A on the disc-shaped residue contacts the grinding tool; (**d**) removal process of the disc-shaped residue when the *h_i_* of the first grain is less than *H_L_*_1*mx*_; and (**e**) morphology of the disc-shaped residue when the workpiece contacts with *D_a_*.

As shown in Figure 8a, based on the motion relationship depicted in Figure 6, when the tool center is at O_2_, point A is removed for the first and only time by the first grain, with a removal height of *H_L_*_1*mx*_; subsequently, point A continues to be ground by the abrasive grains on the end face, with a removal height of *a_p_* minus *H_L_*_1*mx*_, completing one helical cycle of cutting when the peripheral grains on the tool’s end face just lose contact with point A, resulting in a total removal height of *a_p_* for point A. At this time, point B just moves to a position tangent to the edge of the absence zone, starting an identical grinding process cycle to that of point A. The final morphology of the disc-shaped residue is shown in Figure 8b.

Type 2: The protrusion height *h_i_* of the first grain is less than the maximum height *H_L_*_1*mx*_ of the disc-shaped residue. A part of the disc-shaped residue is removed by the first grain, while the rest makes contact friction with the tool’s absence zone, being removed after wear, forming a new contact friction surface.

As shown in Figure 8c, before the tool center moves to O_2_, point A necessarily makes contact with the tool’s end face, with the contact position depending on the protrusion height *h_i_* of the first grain; when the tool’s face makes contact with the workpiece, additional axial force and friction force are formed. As the tool center moves from O_1_ to O_2_, the additional axial force and friction force continuously increase. As shown in Figure 8d, when the tool center reaches O_2_, point A is cut for the first and only one time by the side edge of the first grain, and then it continues to be cut by the grains on the end face until its removal height reaches *a_p_*, completing the cutting process as the peripheral grains on the tool’s end face just lose contact, starting the cycle anew with point B. The morphology of the disc-shaped residue is as depicted in Figure 8e.

## 3. Helical Grinding Experiment Under Central Abrasive Grain Absence on the Tool’s End Face

In order to verify the existence of disc-shaped residues, micro-hole helical grinding experiments were conducted using SiC_p_/Al material. Due to the randomness of the abrasive grain absence at the tool center and the limited protrusion height of abrasive grains (*h_i_*), setting a large single-layer cutting depth (*a_p_*) is challenging, making it difficult to observe obvious disc-shaped residues at the hole bottom. To address these limitations, a specially designed tool with a central edge absence was used to simulate the removal process under the condition of central abrasive grain absence, and relevant cutting experiments were conducted.

**Figure 9:**
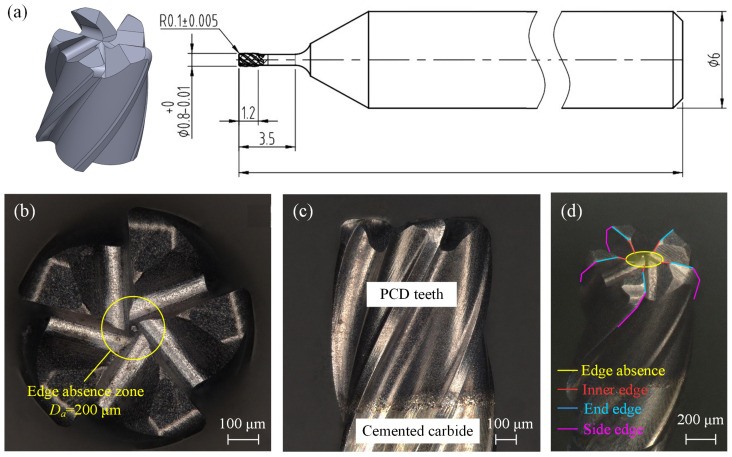
Tool images: (**a**) 3D model and overall dimensions of the cutting edge; (**b**) end face morphology of the tool; (**c**) side view morphology; and (**d**) overall tool tip morphology and the different edges’ distribution.

The geometric model and dimensions of the tool are shown in Figure 9a. The cutting edge has a diameter of 0.8 mm and a total length of 3.5 mm, with the cutting edge material being made of polycrystalline diamond (PCD) and the tool body composed of ultrafine-grain carbide, as shown in Figure 9c. The tool features a five-edge uniform distribution design and was manufactured using an electrochemical grinding process, with a specially sized central grain absence zone. The diameter of this absence zone is shown in Figure 9b,d, and the tool parameters are listed in Table 1.

**Figure 10:**
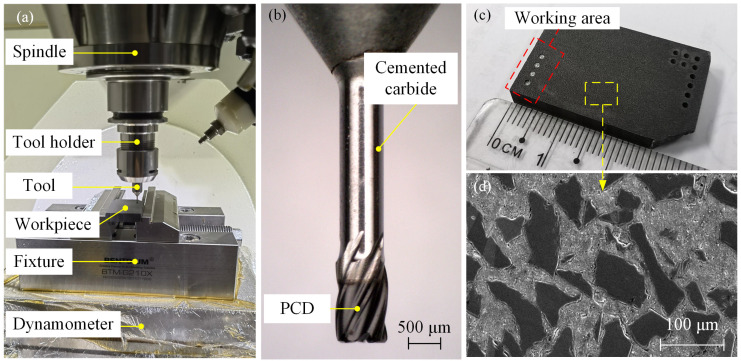
Setup for helical grinding of small holes: (**a**) experimental site; (**b**) five-tooth PCD tool; (**c**) working area on the workpiece; and (**d**) microscopic surface morphology of the workpiece.

The experimental setup is illustrated in Figure 10a, with the tests performed on a Beijing Jingdiao 5-axis machining center (model JDGR-200_A10H). The cutting forces were measured using a Kistler 9257B dynamometer (Kistler, Winterthur, Switzerland). A new tool was used and its figure is shown in Figure 10b. The workpiece material, with a 65% volume fraction SiC_p_/Al, is detailed in Table 2, while its macro- and micromorphology are shown in Figure 10c,d. This composite material, consisting of micron-sized SiC particles as the reinforcing phase and an aluminum alloy matrix, is widely used in electronic packaging applications, such as T/R module and high-power device packaging. Its excellent wear resistance presents significant challenges for grinding. Grinding oil was used throughout the process to ensure proper lubrication and chip removal.

The micro-hole processing parameters are presented in Table 3. To effectively measure the disc-shaped residues and increase their volume, a larger *a_p_* was selected. Given the difficulty of machining this material and its high cutting resistance, a lower feed rate (*v_ft_*) and a higher spindle speed were chosen to control the material removal per edge while ensuring stable tool rotation and avoiding chatter. Since the bottom morphology of the hole needed to be observed, the maximum hole depth was limited to 0.5 mm. Moreover, because the machine performs a final horizontal feed after helical grinding to smooth the bottom surface, the process was interrupted via an emergency stop to preserve the disc-shaped residues, limiting the actual processing depth to approximately 0.3 mm. After completing the experiment, the workpiece was ultrasonically cleaned, and the hole bottom morphology was observed using a Zeiss Gemini 30 electron microscope (Oberkochen, Germany) and an Olympus LEXT OLS5100 3D laser (Shinjuku, Japan) confocal microscope to measure the height of the disc-shaped residues.

### 3.1. Analysis of Hole Bottom Patterns

**Figure 11:**
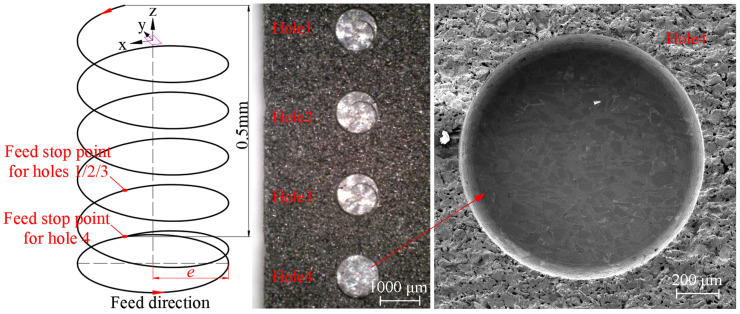
Micro-hole machining morphology and its relationship with the feed depth.

As shown in Figure 11, the bottom morphology of the holes after helical grinding is presented. Holes 1–3 depict the bottom morphology when the tool was stopped by pressing the emergency stop button during the machining process, with a cutting depth of approximately 0.3–0.4 mm. Hole 4 shows the bottom morphology after the completion of the normal grinding process, with a cutting depth of 0.5 mm. From the figure, it is evident that the bottom of Hole 4 is smooth and flat, which is due to the final horizontal feed step that smooths the hole bottom after the helical grinding. This final pass removes the disc-shaped residues via the tool’s outer cutting edges, leaving a smooth surface with no residues. This phenomenon demonstrates that even with a central grain absence tool, good bottom surface quality can be achieved without being affected by the absence zone.

**Figure 12:**
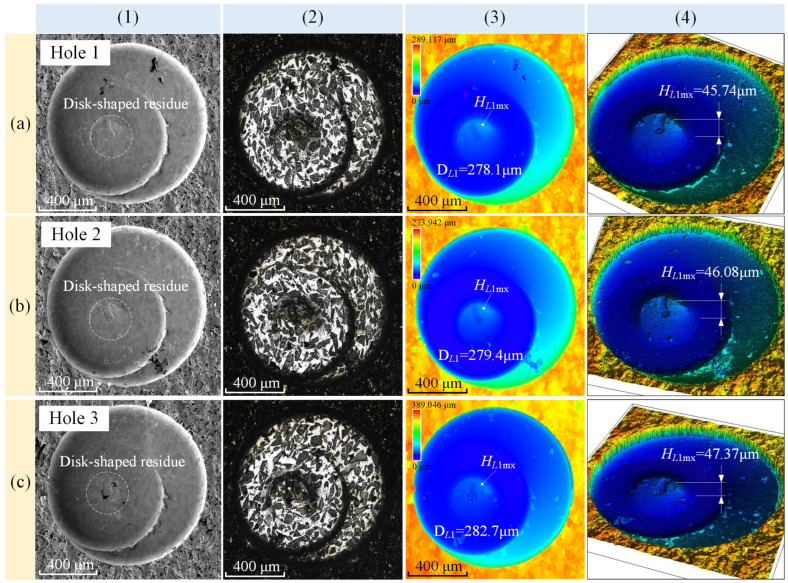
Bottom surface morphology of Hole 1/Hole 2/Hole 3: (**a1**–**a4**) electron microscope, optical images, and 2D/3D depth of field images of Hole 1; (**b1**–**b4**) electron microscope and depth of field microscope images of Hole 2.; and (**c1**–**c4**) electron microscope and depth of field microscope images of Hole 3.

For Holes 1–3, rough bottom surfaces can be clearly observed in Figure 11. After high-resolution electron microscopy and laser confocal microscopy analyses of these three holes, the micro-morphology of the hole bottoms is displayed in Figure 12. Column (1) shows the electron microscopy images, column (2) displays the optical images, and columns (3–4) present the 2D and 3D depth of the field images. From the images in column (3), the diameter of the disc-shaped residue, *D_L_*_1_, was accurately measured, with an average value of 280.1 μm. Additionally, the maximum height difference between the highest point of the disc-shaped residue and the surrounding area, *H_L_*_1*mx*_, was measured, with an average value of 46.40 μm.

**Figure 13:**
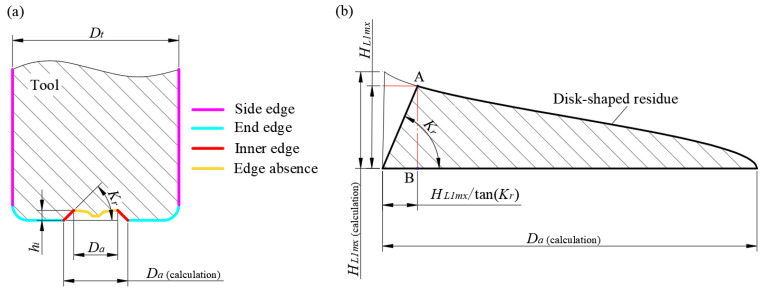
Calculation process for the height of the disc-shaped residues, considering the lead angle of the inner edge. (**a**) edge distribution of the cutting tool; (**b**) cross-section of the disc-shaped residue with a lead angle of the inner edge.

Using the theoretical derivation method outlined in the previous section, the theoretical maximum height of the disc-shaped residue can be calculated as follows. As shown in Figure 13a, since the PCD tool’s inner edge has a lead angle *K_r_*, the inner edge can also be regarded as part of the absence zone. First, the maximum height of the disc-shaped residue *H_L_*_1*mx* (calculation)_ and the diameter of the absence zone *D_a_*_(calculation)_ are determined. After accounting for the lead angle of the inner edge, the corrected disc-shaped residue height *H_L_*_1*mx*_ can finally be obtained.

Figure 13a shows the relationship between the *D_a_* _(calculation)_, *D_a_*, and the inner edge. The actual absence zone diameter *D_a_* is 200 μm, and the projection length of the inner edge on the tool bottom is 40 μm, making the *D_a_*_(calculation)_ 280 μm. Based on the tool parameters and the machining parameters listed in Table 2, using Equation (9), the maximum height of the disc-shaped residue is calculated to be *H_L_*_1*mx* (calculation)_ = 46.58 μm. After considering the lead angle of the inner edge and using the geometric relationship illustrated in Figure 13, the corrected maximum height of the disc-shaped residue can be estimated using Equation (6).

(10) $$H_{L1mx}=\frac{a_{p}}{\pi }\arctan\frac{\sqrt{\frac{D_{a(\mathrm{calculation})}^{2}}{4}-{\left(\frac{H_{L1mx}}{\tan K_{r}}\right)}^{2}}}{e+\frac{H_{L1mx}}{\tan K_{r}}}$$

Solving the transcendental equation numerically: by substituting the relevant parameters and *K_r_* = 60° into Equation (10), the theoretical maximum height of the disc-shaped residue is calculated to be 42.22 μm.

**Figure 14:**
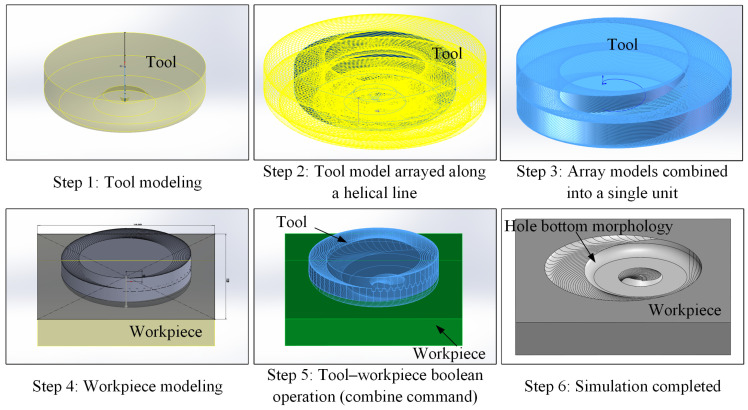
Simulation process for the disc-shaped residues (version: Premium 2020 SP0.0).

To investigate the undeformed chip morphology of the side edge grains and end edge grains during the helical grinding process and determine the cutting removal depth of abrasive grains in different edge zones, a kinematic simulation of the cutting process was conducted. As shown in Figure 14, the kinematic simulation was conducted using SolidWorks 3D modeling software (Version: Premium 2020 SP0.0). Both the tool and workpiece were assumed to be rigid bodies, with material properties such as the type, density, strength, and Poisson’s ratio being neglected—only rigid contact and resulting bottom surface morphology were considered. The specific steps of the simulation process were as follows. To ensure stable software operation, the model was scaled up to 10 times the actual tool size. Step 1, establish the geometric model of the tool under its rotation state according to the actual dimensions. Step 2, apply the curve-driven array command along the helical feed path, with 80 array units and 1.5 helical turns. Step 3, combine all the arrayed tool models into a new integrated model. Steps 4–5, create the 3D geometry of the workpiece, and perform a Boolean operation with the tool model from Step 3, subtracting the overlapping volumes to achieve the final simulation results shown in Step 6.

**Figure 15:**
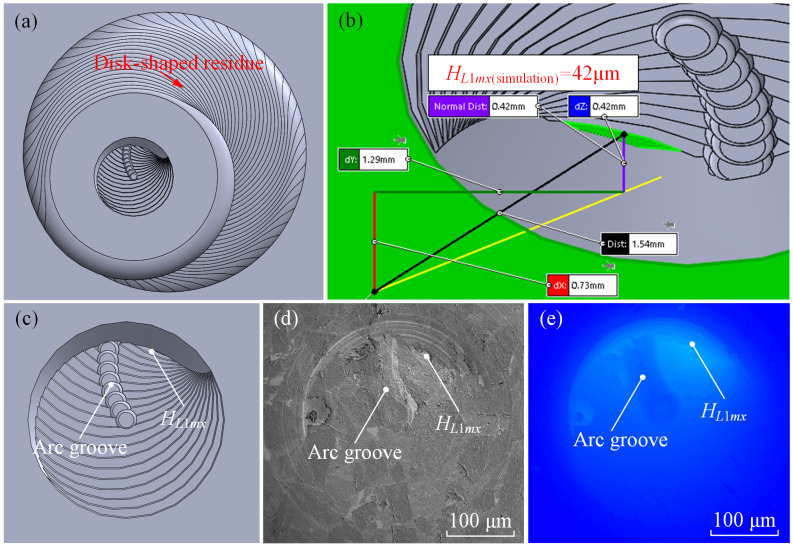
Simulated and actual morphology of the disc-shaped residues: (**a**–**c**) kinematic simulation morphology, (**d**) electron microscope image, and (**e**) laser confocal microscope image.

As presented in Figure 15, the morphology of the disc-shaped residue obtained from the simulation is displayed. Figure 15a shows the overall top view, Figure 15b presents the measurement data of the maximum height of the disc-shaped residue, and Figure 15c shows the overall simulation morphology. Figure 15d,e depicts the actual electron microscopy and depth of field images of the disc-shaped residue. From Figure 15b, the maximum height difference between the simulated residue and the bottom surface, divided by the magnification factor (10×), yields a simulated *H_L_*_1*mx* (simulation)_ of 42.00 μm. By comparing Figure 15c–e, it is evident that the simulated residue morphology closely matches the actual morphology obtained in the experiment. However, due to chip stacking, tool edge roughness, and other factors in the actual machining process, the residue morphology appears slightly irregular. Nonetheless, the depth-of-field intensity image in Figure 15e clearly shows an elevated region at the center of the tool, matching the simulated residue morphology. The arc grooves on the residue surface are formed by the tool’s central protrusion contacting and cutting/rubbing the disc-shaped residue. However, these grooves do not intersect the maximum height point of the residue, so they do not affect the measurement of the maximum height. The formation of the grooves also suggests that when the protrusion height of the tool’s absence zone is insufficient, contact friction with the disc-shaped residue can occur. Therefore, extra care must be taken in tool design and parameter selection to ensure the maximum height of the disc-shaped residue is lower than the height of the inner cutting edge, with an additional safety margin to prevent interference and friction between the residue and the tool’s bottom absence zone.

**Figure 16:**
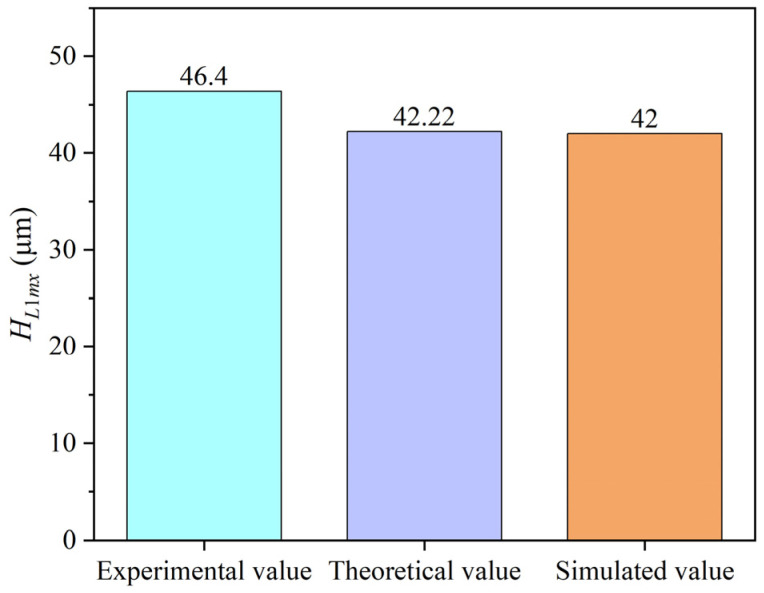
Comparison of the maximum height of the disc-shaped residues obtained by different methods.

As shown in Figure 16, the actual maximum height of the disc-shaped residue, the theoretical maximum height, and the simulated maximum height are compared. The theoretical and simulation results are quite close, while the actual values are significantly higher, mainly due to micro-roughness, burrs, or chip stacking during material machining, which affect the measurement of the maximum height. The maximum error between the actual and theoretical/simulation results is 9–9.1%, confirming the accuracy of the theoretical derivation in this study. These findings demonstrate that disc-shaped residues do indeed form during helical grinding with the absence of abrasive grains at the tool center, and these residues are removed by the first effective abrasive grain surrounding the absence zone, which in the validation experiments was removed by the inner cutting edge with a similar function.

### 3.2. Cutting Force Analysis

**Figure 17:**
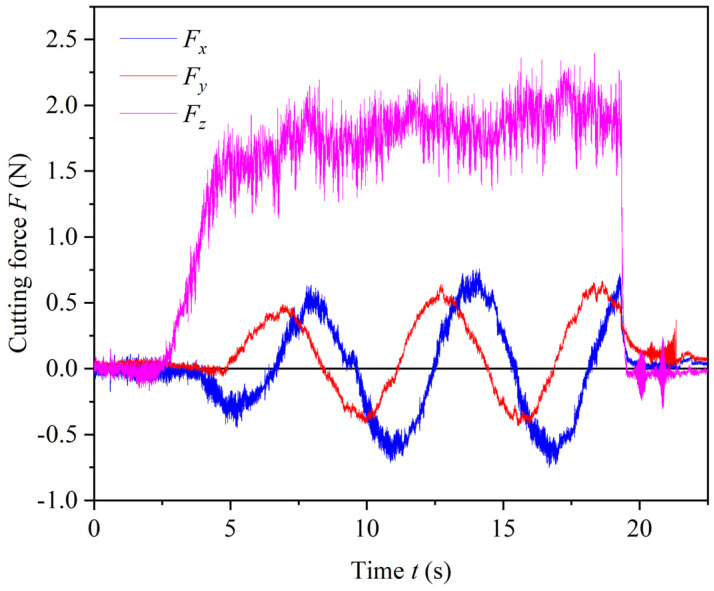
Cutting force of Hole 3.

As shown in Figure 17, the cutting force variations during the machining process of Hole 3 are illustrated. It can be observed that *F_x_* and *F_y_* alternate in a fluctuating manner, consistent with the typical cutting force characteristics in helical milling/grinding. The maximum value of *F_x_* is 0.76 N, *F_y_* reaches a maximum of 0.67 N, and *F_xy_* has a maximum of 0.87 N. *F_z_* exhibits small fluctuations, with a gradual upward trend, but overall, the cutting forces remain relatively low, with a maximum *F*_z_ of 2.40 N. The maximum resultant cutting force during the entire process is 2.49 N. This indicates that, due to the multi-blade design of the tool and the incorporation of optimal rake and clearance angles along with the helical angle, the cutting performance is enhanced. Additionally, the absence of cutting edges in the center improves the overall cutting speed of the tool’s bottom edge, resulting in lower cutting forces.

## 4. Conclusions

This study investigates helical grinding under conditions of central abrasive grain absence and graphically explores the formation of undeformed chips. The key findings are as follows:

1. The research demonstrates that all grinding tools, regardless of the manufacturing method or grain size, exhibit a central grain absence potential. Larger grain sizes lead to a more pronounced and extensive absence area.
2. Analysis of helical grinding with a central grain absence revealed specific residual patterns at the hole’s bottom. These patterns depend on the absence zone’s diameter relative to the tool’s eccentricity. Analytical models were developed to describe the heights of the disc-shaped residues.
3. This study explored the removal mechanisms of cylindrical and disc-shaped residues. Cylindrical residues, which cannot be removed by grinding and cause interference, should be avoided. For disc-shaped residues, the removal process varies based on the protrusion height of the first grain. When the protrusion height is sufficient, the first grain cuts away the residue; when insufficient, the residue is partially removed through contact and friction with the absence zone.
4. In the experimental validation, an innovative micro-PCD multi-blade milling–grinding tool with a central edge absence was designed and manufactured. This tool was used for cutting experiments on 1.1 mm diameter holes in SiC_p_/Al (65% volume fraction) material. By stopping the tool during machining, the bottom morphology of the hole was preserved. Microscopic observation confirmed that disc-shaped residues form at the hole bottom when the tool’s center lacks abrasive grains. The residue’s shape remains constant but shifts with the tool’s center movement. The experimentally measured maximum height of the disc-shaped residue was compared with theoretical and simulation results, confirming the accuracy of the theoretical research.

These findings contribute to the theoretical framework of helical grinding–milling and offer valuable theoretical and practical insights for machining micro-holes with tools featuring central abrasive grain absence in hard-to-machine ceramics.

## Figures and Tables

**Table 1:** Tool parameters.

Item	Value
Number of edges	5
Diameter	$0.8_{-0.01}^{+0}$ mm
PCD length	1.2 mm
Cutting edge overhang	3.5 mm
Overall length	40 mm
Diameter of central edge absence zone	200 μm
Inner edge height	60 μm
Lead angle of the inner edge	60°
Tool tip arc radius	R0.1±0.005 mm

**Table 2:** Composition and properties of SiC_p_/Al [32].

Parameter	Value
Average SiC particle size (μm)	80.0
SiC volume fraction (%)	65.0
Thermal conductivity (W/mK, 373.15 K)	73.0
Coefficient of thermal expansion (10^−6^/K)	12.0
Density (g/cm^3^)	3.0
Elastic modulus (GPa)	188.0
Poisson’s ratio	0.3

**Table 3:** Machining parameters.

Spindle Speed *n* (rpm)	Horizontal Feed Rate *v_ft_* (mm/min)	Eccentricity *e* (mm)	Hole Diameter *D_h_* (mm)	Single-Layer Cutting depth *a_p_* (mm)	Hole Depth (mm)
30,000	10	0.15	1.1	0.1	0.3–0.5

## Data Availability

The original contributions presented in the study are included in the article, further inquiries can be directed to the corresponding author.
